# chi-miR-99b-3p Regulates the Proliferation of Goat Skeletal Muscle Satellite Cells In Vitro by Targeting *Caspase*-*3* and *NCOR1*

**DOI:** 10.3390/ani12182368

**Published:** 2022-09-11

**Authors:** Rongrong Liao, Yuhua Lv, Jianjun Dai, Defu Zhang, Lihui Zhu, Yuexia Lin

**Affiliations:** Institute of Animal Husbandry and Veterinary Science, Shanghai Academy of Agricultural Sciences, Shanghai 201106, China

**Keywords:** chi-miR-99b-3p, proliferation, apoptosis, goat, *Caspase*-*3*, *NCOR1*

## Abstract

**Simple Summary:**

In this study, we investigated the role of chi-miR-99b-3p in goat skeletal muscle satellite cells (SMSCs). We confirmed that chi-miR-99b-3p promoted proliferation through targeting *Caspase-3* and nuclear receptor corepressor 1, and inhibiting the intrinsic apoptosis-related genes in SMSCs, whereas inhibition of chi-miR-99b-3p had the opposite effect. Furthermore, integrative transcriptomic analysis revealed that overexpression of chi-miR-99b-3p induced various differentially expressed (DE)-gene-associated processes. In addition, 47 dysregulated miRNAs, including 16 upregulated and 31 downregulated miRNAs, which were involved in the biological pathways associated with the DE genes, were identified. Our study demonstrated that chi-miR-99b-3p was an effective facilitator of goat SMSCs. This study is helpful for future studies in skeletal muscle development.

**Abstract:**

We previously found that chi-miR-99b-3p was highly expressed in the skeletal muscle of 7-month-old (rapid growth period) goats and speculated that it may be associated with muscle development. To further investigate the role of chi-miR-99b-3p in goats, we found that chi-miR-99b-3p acted as a myogenic miRNA in the regulation of skeletal muscle development. Dual-luciferase reporter assays, qRT-PCR, and Western blot results confirmed that *Caspase-3* and nuclear receptor corepressor 1 were direct targets for chi-miR-99b-3p as their expression was inhibited by this miR. Cell proliferation and qRT-PCR assays showed that chi-miR-99b-3p promoted proliferation through relevant targets and intrinsic apoptosis-related genes in goat skeletal muscle satellite cells (SMSCs), whereas inhibition of chi-miR-99b-3p had the opposite effect. Furthermore, integrative transcriptomic analysis revealed that overexpression of chi-miR-99b-3p induced various differentially expressed (DE) genes mainly associated with the cell cycle, relaxin signaling pathway, DNA replication, and protein digestion and absorption. Notably, most of the cell-cycle-related genes were downregulated in SMSCs after miR-99b-3p upregulation, including the pro-apoptosis-related gene *BCL2*. In addition, 47 DE miRNAs (16 upregulated and 31 downregulated) were determined by Small RNA-sequencing in SMSCs after chi-miR-99b-3p overexpression. Based on the KEGG enrichment analysis, we found that these DE miRNAs were involved in the biological pathways associated with the DE genes. Our study demonstrated that chi-miR-99b-3p was an effective facilitator of goat SMSCs and provided new insights into the mechanisms by which miRNAs regulate skeletal muscle growth in goats.

## 1. Introduction

As an economically important agricultural animal, the goat provides humans with high-quality and low-cholesterol mutton. Muscle is an important tissue in goats, and constitutes the main form of mutton. Therefore, the identification of methods to improve goat muscle development and growth is an important economic problem. The formation of animal muscle tissue mainly depends upon the accumulation of proteins and the coordination of muscle cell proliferation, differentiation, and apoptosis, and includes three main stages: myotube formation, myotube differentiation into muscle fibers, and muscle fiber growth and maturation [1]. Skeletal muscle satellite cells (SMSCs) have stem-cell-like properties and are involved in postnatal skeletal muscle regeneration and growth [2,3,4]. Due to their myogenic and adipogenic differentiation capacity, SMSCs have become a suitable model for investigating postnatal muscle growth and intramuscular adipogenesis [5]. The development of SMSCs is a complex and delicate process that is regulated by many factors, including muscle cell migration, proliferation, differentiation, and apoptosis, and needs the coordinated control of multiple signal switches. For example, knockdown of *MUSTN1* inhibited the proliferation and differentiation of chicken SMSCs and promoted the apoptosis of SMSCs, while overexpression of *MUSTN1* showed the opposite effect [6], demonstrating the potential relationship among apoptosis, proliferation, and differentiation of SMSCs.

MicroRNAs (miRNAs) represent endogenous, small noncoding RNAs with a length of 20~25 bp, and are widely found in many species. They are highly evolutionarily conserved and play important roles in the complex and dynamic network that regulates development. miR-487b-3p downregulated *IRS1* expression to suppress goat myoblast cell proliferation and differentiation through the IRS1/PI3K/Akt pathway [4], miR-181a could suppress SMSC proliferation [2], and miR-223-3p was involved in muscle regeneration via the regulation of inflammatory cytokine secretion in the skeletal muscle microenvironment [7].

miR-99b-3p is evolutionarily conserved among different species. However, few studies have investigated the role of miR-99b-3p in goats, as most have been performed on human cancer cell proliferation. For example, evidence shows that miR-99b-3p can target protocadherin 19 to promote hepatocellular carcinoma metastasis and cell proliferation [8]. Furthermore, Hsa-miR-99b-3p is considered a novel prognostic marker for oral cancer, as its increased expression showed a trend toward the advanced tumor stage. In addition, high hsa-mir-99b-3p expression levels were related to better survival [9]. Here, miR-99b-3p exerted an antiproliferative effect and arrested gastric cancer cells in the S phase of the cell cycle by inhibiting cell viability and targeting *HoxD3*, which is upregulated in GC cell lines [10]. In addition, miR-99b-3p was reported to contribute to angiotensin-II-induced cardiac fibrosis in mice [11].

Studies have shown that miRNAs expressed in human skeletal muscle are associated with muscle size, strength, and mass. Muscle strength and cross-sectional area were positively correlated with miR-99b-5p expression in middle-aged men [12]. In our previous study, we found that chi-miR-99b-3p was highly expressed in the muscle tissue of Chongming white goats during the fast-growing period (7 months old), while it was downregulated in postweaning goats, which caused growth retardation in early weaned lambs [13,14]. Therefore, we speculated that chi-miR-99b-3p might be related to muscle development [13]. Here, we systematically investigated the potential role of chi-miR-99b-3p in goat SMSCs. Our findings help with understanding the function of miRNAs in skeletal muscle development and provide a theoretical basis for the molecular breeding of goats.

## 2. Materials and Methods

### 2.1. Cell Culture and Cell Transfection

Isolated goat SMSCs (separated from Anhui white goats) were a kind gift from Dr. Yinghui Ling (College of Animal Science and Technology, Anhui Agricultural University, Hefei, China). Detailed isolation steps for SMSCs can be found in a previous study [3]. The 9th to 12th generations of satellite cells were used in this study. The SMSCs were cultured in DMEM/F12 (Gibco, Grand Island, NY, USA) growth media containing 20% FBS (Gibco, Grand Island, NY, USA) and 1% penicillin/streptomycin (Gibco, Grand Island, NY, USA). The chi-miR-99b-3p mimic, negative control (NC mimic), chi-miR-99b-3p inhibitor were transfected into cells using Entranster^TM^-R4000 reagent (Engreen, Beijing, China) according to the manufacturer’s protocol. Cells were collected for further analysis at the indicated times. Oligonucleotides were designed and synthesized by Shanghai GenePharma Co., Ltd. (Shanghai, China), and their sequences can be found in Table S1. For cell-differentiation-related analysis, SMSCs were first cultured in growth media until the cells grew to about 80% density. Differentiation medium, including 2% FBS (Gibco, Grand Island, NY, USA) and 1% penicillin/streptomycin (Gibco, Grand Island, NY, USA), was used to induce cell differentiation. Cells collected 1 d after the addition of differentiation medium were used as the control.

### 2.2. RNA Isolation and qRT-PCR

Total RNA was isolated using TRIzol reagent (Invitrogen, Waltham, MA, USA) and determined in an ND-2000 spectrophotometer (Thermo Scientific, Wilmington, DE, USA), and the qRT-PCR was performed as previously described [13]. Briefly, the expression levels of miRNAs and mRNA were determined using qRT-PCR performed on an ABI QuantStudio 5 PCR System (Applied Biosystems, Foster City, CA, USA) using SYBR Green PCR Master Mix (Fast Start Universal SYBR Green Master; Roche, Basel, Switzerland). Primer sequences are listed in Table S2. Glyceraldehyde 3-phosphate dehydrogenase was used as an endogenous control gene for mRNA, and U6 for miRNA. Relative quantitative levels were calculated based on the 2^−ΔΔCt^ method [4].

### 2.3. Western Blotting

Total protein was purified from satellite cells using radioimmunoprecipitation assay (RPMI) buffer (Beyotime, Shanghai, China), and electrophoresed on an 8−12% sodium dodecyl sulfate polyacrylamide gel, and transferred to polyvinylidene fluoride membranes (Millipore Sigma, Burlington, MA, USA). Next, the membranes were blocked with 5% (*w*/*v*) bovine serum albumin and incubated with primary antibodies against *Caspase-3* (GB11767c; Servicebio, Wuhan, China), *Caspase-7* (27155-1-ap; Proteintech, Chicago, IL, USA), *Caspase-9* (GB11053-1; Servicebio, Wuhan, Hubei, China), *BCL2* (BF9103; Affinity Biosciences, OH, USA), and β-actin (8227; Abcam, Cambridge, MA, USA), and visualized using an ECL Western blot detection kit (Thermo Scientific, Rockford, IL, USA) and ImageQuant LAS 4000 (GE Healthcare, Piscataway, NJ, USA).

### 2.4. Dual Luciferase Reporter Assay

Wild-type and mutated sequences of the 3ʹUTR of chi-miR-99b-3p were synthesized and cloned into psiCHECK-2 plasmid (Promega, Madison, WI, USA). Subsequently, the plasmids were cotransfected with the miRNA mimic or negative control miRNA into HEK293T cells. At 30 h post-transfection, luciferase activity was detected using a Dual-Glo^®^ Luciferase Assay System (Promega) according to the manufacturer’s instructions.

### 2.5. Cell Proliferation Assays 

Cells were seeded into a 96-well plate and cell proliferation was measured using a cell counting kit-8 (CCK-8) reagent (Beyotime, Shanghai, China) 30 h after transfection according to the manufacturer’s protocol.

### 2.6. Transcriptome Sequencing Analysis

Total RNA from the miRNA-treated cells was purified with beads containing oligo (dT) and then used to generate cDNA with adapter addition to generate cDNA fragments by PCR. The products were sequenced on a BGISEQ-500 system (BGI, Shenzhen, China). Clean reads were obtained by removing reads containing adapter, unknown ploy N, and low-quality reads from the raw data. Bowtie 2 (V2.2.5) (http://bowtie-bio.sourceforge.net/bowtie2/index.shtml, Baltimore, USA) (accessed on 2 March 2022) was used to align the clean reads to produce sets. Differentially expressed genes (DEGs) were quantified using the cutoff |log2 fold change| > 1, *p* ≤ 0.05 by DESeq2 (https://bioconductor.org/packages/release/bioc/html/DESeq2.html) (accessed on 2 March 2022). The pathway enrichment analysis was performed by searching against the KEGG database. Significant terms with correction and *p* ≤ 0.05 were considered statistically significant.

### 2.7. Small RNA Sequencing Analysis

Total RNA from the miRNA-treated cells was extracted using TRIzol reagent (Invitrogen, Carlsbad, CA, USA) and qualified using an Agilent 2100 Bioanalyzer (Thermo Fisher Scientific, Waltham, MA, USA). Libraries were prepared with a Small RNA Cloning Kit (TaKaRa, Dalian, China) and sequenced on a BGISEQ-500 platform (BGI, Shenzhen, China). Clean tags were mapped against miRBase for miRNA with Bowtie 2 (V2.2.5). MiRanda (v2.041) (https://www.cs.kent.ac.uk/people/staff/dat/miranda/downloads/cygwin/) (accessed on 2 March 2022), RNAhybrid (v2.1) (https://bibiserv.cebitec.uni-bielefeld.de/rnahybrid, Bielefeld, Germany) (accessed on 2 March 2022), and TargetScan (v5.0) (https://www.targetscan.org/vert_50/, Cambridge, USA) (accessed on 2 March 2022) were used to predict the target genes of miRNAs. Only overlapping genes were selected as candidate targets of the DE miRNAs. Then, the candidate targets were further screened by the DE genes above, and only genes recognized by the DE genes and predicted targets were considered as candidate genes targeted by the DE miRNAs. To annotate gene functions, all target genes were aligned against the Kyoto Encyclopedia of Genes (KEGG) database using phyper in R software (v3.3.1) (https://www.r-project.org/) (accessed on 2 March 2022). Differential expression analysis was performed using DEGseq (v1.4.5) (https://www.bioconductor.org/packages/release/bioc/html/DEGseq.html) (accessed on 2 March 2022) under the standard of |log2 fold change | ≥ 1 and Q value ≤ 0.001. Sequencing data were deposited into the NCBI SRA database with accession number PRJNA844520. The same total RNA from miRNA treated cells with triplicate biological replicates was used for transcriptome and small RNA-sequencing analysis.

### 2.8. DEmiRNA and DEG Interaction Analysis

The interaction network between the DE miRNAs and the DE genes was constructed using Cytoscape v.3.7.2 software (https://cytoscape.org/release_notes_3_7_2.html) (accessed on 2 March 2022) as previously described [13].

### 2.9. Statistical Analysis

One-way analysis of variance and Tukey’s test were used for statistical analysis. Results are presented as the mean ± SEM of at least triplicate replicates. For statistically significant differences, *p* < 0.05 was required. All statistical analyses were carried out using SPSS 24.0 software (https://www.ibm.com/support/pages/downloading-ibm-spss-statistics-24) (accessed on 2 March 2022).

## 3. Results

### 3.1. Caspase-3 and NCOR1 Are Targeted by chi-miR-99b-3p

**Figure 1:**
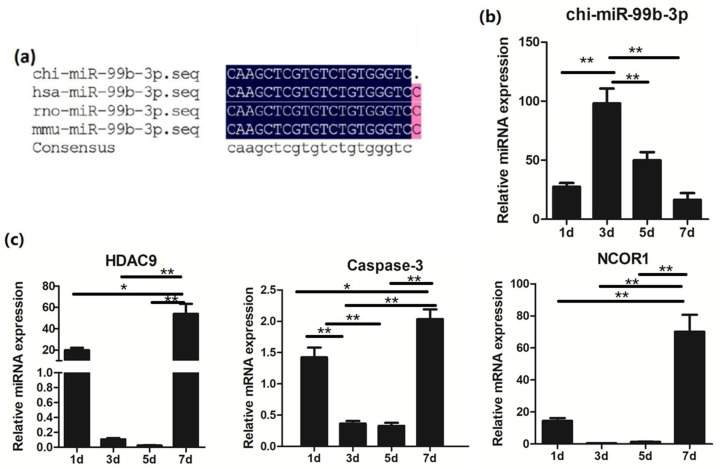
Relative expression of chi-miR-99b-3p and potential targets during the differentiation of SMSCs. (**a**) chi-miR-99b-3p was conserved across species. (**b**) Relative expression of chi-miR-99b-3p during the differentiation of SMSCs. (**c**) mRNA expressions of selected targets during the differentiation of SMSCs. All results are presented as means ± SEM. *n* = 3. * *p* < 0.05; ** *p* < 0.01.

To determine the molecular function of chi-miR-99b-3p in muscle development, we first confirmed the mature sequence of chi-miR-99b-3p, and found that it was conserved in different species (Figure 1a); then, the potential targets of chi-miR-99b-3p were predicted by miRanda, Pita, and TargetScan software, as shown in Table S3. A total of 35 genes related to the development of skeletal muscle were predicted to be targeted by chi-miR-99b-3p. Among them, *Caspase-3*, histone deacetylase 9 (*HDAC9*), and nuclear receptor corepressor 1 (*NCOR1*) were the predicted targets of chi-miR-99b-3p. To further elucidate the relationship between chi-miR-99b-3p and the above three potential targets, we measured the expressions of chi-miR-99b-3p, *Caspase*-*3*, *HDAC9*, and *NCOR1* on different days post-culture of SMSCs during their differentiation (Figure 1b,c). The results showed that the endogenous expression of chi-miR-99b-3p increased, and the mRNA expressions of *Caspase*-*3*, *HDAC9*, and *NCOR1* decreased at 3 and 5 d of SMSC differentiation (Figure 1c). The expressions of *Caspase-3*, *HDAC9*, and *NCOR1* were significantly increased on day 7, with no significant difference in chi-miR-99b-3p expression between 1 d and 7 d of SMSC differentiation (Figure 1b).

**Figure 2:**
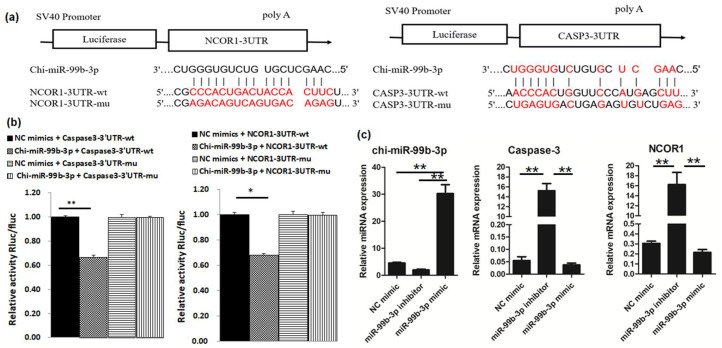
*Caspase-3* and *NCOR1* are the target genes of chi-miR-99b-3p. (**a**) Diagram depicting the binding sites of chi-miR-99b-3p on the 3ʹ-UTR of *Caspase-3* and *NCOR1* (highlighted in red). (**b**) Detection of relative luciferase activity. wt, wild-type. mut, mutant-type. All results are presented as means ± SEM. *n* = 3. * *p* < 0.05; ** *p* < 0.01. (**c**) Overexpression or suppression of chi-miR-99b-3p impacted the expressions of *Caspase*-*3* and *NCOR1* at the mRNA level.

We then used a vector psiCHECK-2 to construct 3′-UTR vectors and the corresponding mutant vectors for the potential target genes *NCOR1*, *HDAC9*, and *Caspase*-*3*. As shown in Figure 2a,b, enhanced chi-miR-99b-3p significantly inhibited the luciferase activity of NCOR1-3′-UTR-WT and CASP3-3′-UTR-WT (*p <* 0.01), but not of HDAC9 NCOR1-3′-UTR-WT (Figure S1), indicating that *NCOR1* and *CASP-3* were directly targeted by chi-miR-99b-3p. Furthermore, the levels of chi-miR-99b-3p and its target genes (*Caspase-3* and *NCOR1*) were measured at 30 h in goat skeletal muscle cells after transfection with a chi-miR-99b-3p mimic or inhibitor, and revealed that enhanced chi-miR-99b-3p expression repressed the mRNA expression of *Caspase*-*3* and *NCOR1*, whereas they were increased after the transfection with a chi-miR-99b-3p inhibitor (Figure 2c).

### 3.2. chi-miR-99b-3p Targets Caspase-3 and NCOR1 to Regulate Cell Proliferation and Differentiation

**Figure 3:**
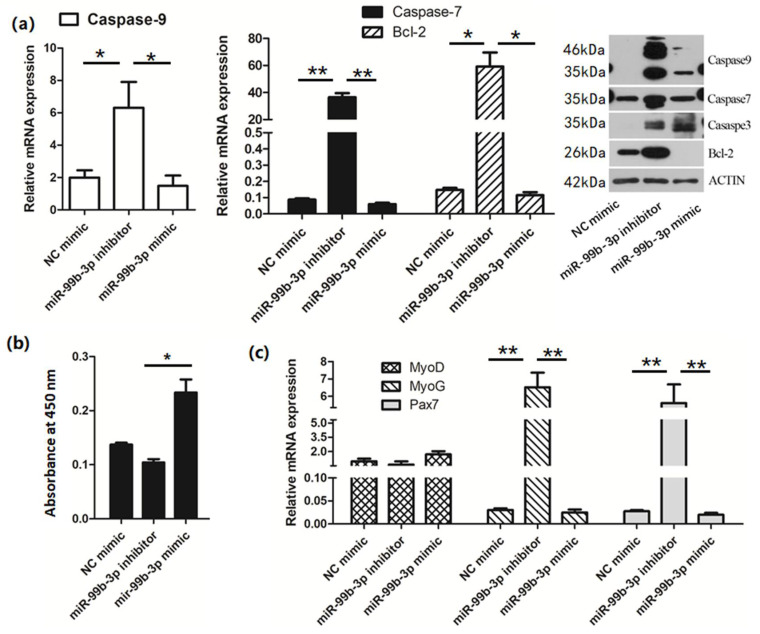
chi-miR-99b-3p regulates cell proliferation and differentiation via the intrinsic apoptotic pathway. (**a**) mRNA and protein levels of apoptosis-related genes after transfection with chi-miR-99b-3p mimic or inhibitor. (**b**) Cell counts detected by CCK-8 analysis. (**c**) mRNA levels of differentiation-related genes after transfection with chi-miR-99b-3p mimic or inhibitor. * *p* < 0.05; ** *p* < 0.01. Original Figure 3a in Figure S2.

Compared with NC, the mRNA and protein expressions of the apoptosis-related genes *Caspase-9*, *7*, and *BCL2* were all successfully downregulated by the miR-99b-3p mimic, but were activated after transfection with the miR-99b-3p inhibitor (Figure 3a). The effects of cell viability and cell-differential-related gene expression at 30 h post-transfection were further analyzed (Figure 3b,c). Overexpression of miR-99b-3p significantly increased cell viability and inhibited the protein levels of *BCL2*, while the mRNA expression levels of apoptosis-related genes *Caspase-9*, *Caspase-7*, and *BCL2* were significantly increased in SMSCs after chi-miR-99b-3p downregulation compared with the control mimic-treated SMSCs (Figure 3a). Additionally, the mRNA expression levels of *MyoG* and *Pax7* were significantly increased in chi-miR-99b-3p-inhibited cells (Figure 3c).

### 3.3. mRNA Profile Analysis after Upregulation of chi-miR-99b-3p

**Figure 4:**
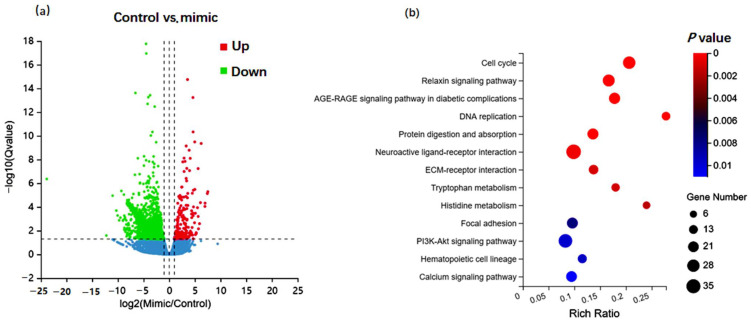
Differentially expressed mRNAs after chi-miR-99b-3p up-regulation. (**a**) A volcano plot representing DEGs after chi-miR-99b-3p overexpression (**b**) Functional enrichment analysis of DE genes.

Using mRNA sequencing analysis, 1490 DE genes were obtained |log2 fold change |≥1 and Q value ≤ 0.001 in miR-99b-3p mimic-transfected cells compared with the NC miRNA-treated cells (Figure 4a). Further functional analysis of these DE genes showed that after overexpression of chi-miR-99b-3p, the DEGs in the skeletal muscle cells were mainly involved in the cell cycle, relaxin signaling pathway, DNA replication, and protein digestion and absorption (Figure 4b), demonstrating that chi-miR-99b-3p was mainly involved in cell cycle regulation. In addition, the DE genes associated with the cell cycle process are listed in Table S4. Most of the cell-cycle-related genes were downregulated in SMSCs after chi-miR-99b-3p upregulation; among them, the pro-apoptosis-related gene *BCL2*, which is demonstrated to be downregulated in Figure 3a, was decreased in chi-miR-99b-3p upregulated cells based on sequencing data. However, we did not see downregulation of *Caspase-3* or *NCOR1* in the SMSCs after chi-miR-99b-3p upregulation, based on the filter condition of |log2 fold change | ≥ 1 and Q value ≤ 0.001. In addition, we also found 34 genes, including *SH2D4A*, *ABCB10*, *B3GNT6*, *OPCML*, and *SPATA13*, predicted to be candidate targets of chi-miR-99b-3p, which were downregulated in the cells after miR-99b-3p upregulation (Table S5).

### 3.4. miRNA Profile Analysis after chi-miR-99b-3p Upregulation

**Figure 5:**
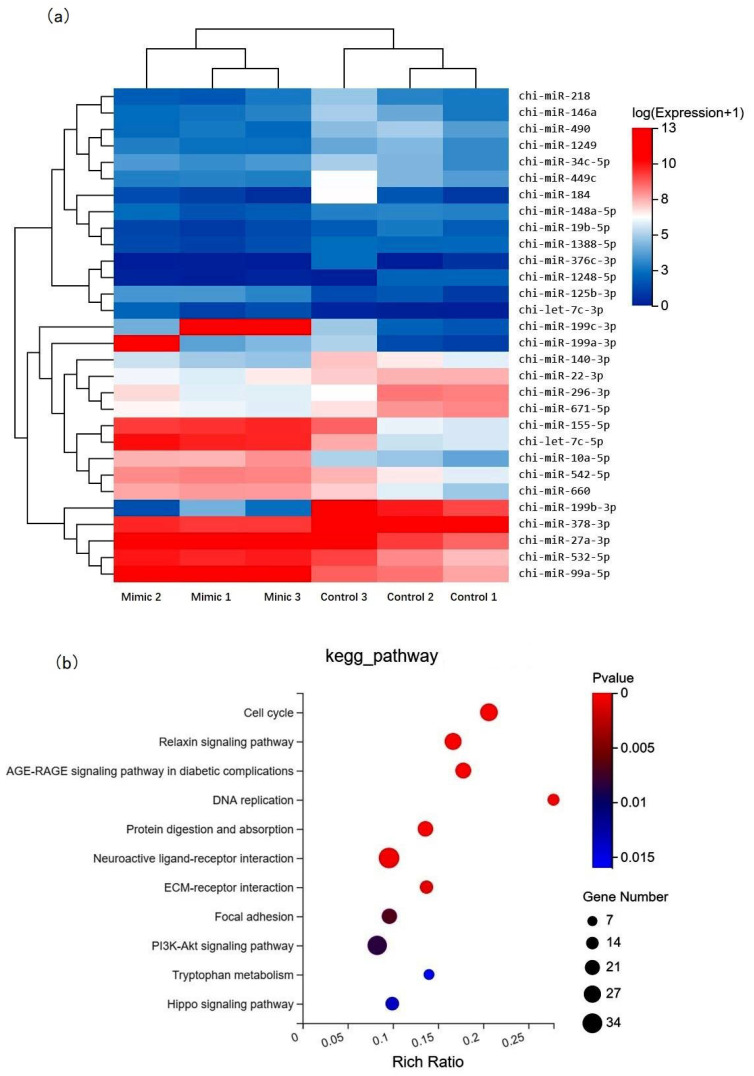
Differentially expressed miRNA expression in SMSCs after miR-99b-3p overexpression. (**a**) Heat map of known DE miRNAs in SMSCs after chi-miR-99b-3p overexpression. (**b**) KEGG functional analysis DE miRNA in SMSCs after chi-miR-99b-3p upregulation.

To evaluate the potential miRNAs influenced by chi-miR-99b-3p upregulation, small RNA-sequencing analysis was performed, and the DE miRNAs are shown in Table S6. Here, chi-miR-99b-3p upregulation induced 47 DE miRNAs, of which 16 were upregulated and 31 downregulated, including 30 known mature miRNAs (Figure 5a) and 17 novel miRNAs. Among them, chi-miR-199c-3p, chi-miR-199a-3p, chi-let-7c-3p, chi-let-7c-5p, chi-miR-10a-5p, chi-miR-125b-3p, and chi-miR-99a-5p represented the seven most highly expressed miRNAs in SMSCs after chi-miR-99b-3p upregulation. We next explored the potential targets of the DE miRNAs by screening the DE genes mentioned above, and overlapping genes were considered as candidate genes targeted for the DE miRNAs (Table S7), and were subsequently used for KEGG analysis. Further functional analysis revealed that these DE miRNAs were involved in the cell cycle, relaxin signaling pathway, DNA replication, and protein digestion and absorption (Figure 5b), and this was consistent with the results seen for the DE genes (Figure 4b).

### 3.5. Integration Analysis of the DE miRNAs and DEGs

**Figure 6:**
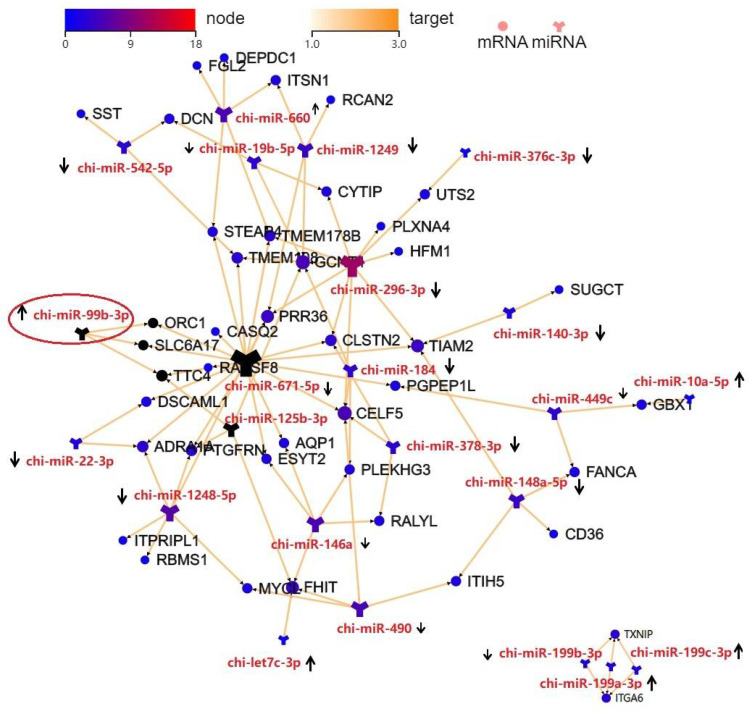
miRNA–mRNA interaction network in SMSCs after miR-99b-3p overexpression. DE miRNAs and mRNAs were considered.

As shown in Figure 6, most miRNAs had multiple potential target genes, while different miRNAs could regulate the same target. For example, miR-671-5p was the regulator of *TTC4*, *CASQ2*, *ORC1*, *SLC6A17*, and *AQP7*; whereas miR-199a-3p, miR-199b-3p, and miR-199c-3p, which belong to the miR-199 cluster, could regulate the expressions of *ITGA6* and *TXNIP*. In addition, chi-miR-99b-3p could regulate *ORC1*, *SLC6A17*, and *TTC4*, which were downregulated in SMSCs after miR-99b-3p overexpression (Table S3).

## 4. Discussion

Skeletal muscle is the most abundant tissue in the body and possesses remarkable regenerative capacity. It consists of a diversity of cell types including multinucleated myofibers, SMSCs, endothelial cells, pericytes, mesenchymal progenitor cells, and immune cells [15]. SMSCs participate in postnatal muscle growth and regeneration in animals, which has been shown to be regulated by miRNAs [2,16]. For example, BMP2 is targeted by miR-378 to regulate the proliferation of sheep myoblast [17] and inhibit apoptosis in skeletal muscle [16]. miR-181a regulates the proliferation and differentiation of Hu sheep SMSCs by directly targeting YAP1 [2]. Here, we found that miR-99b-3p was able to promote cell proliferation and inhibit apoptosis in Anhui white goat SMSCs by blocking the intrinsic apoptosis pathway via targeting *caspase-3* and *NCOR1*. Moreover, integrated mRNA–miRNA analysis revealed that the overexpression of miR-99b-3p upregulated several cell-cycle-associated miRNAs that regulate the cell cycle in SMSCs. These results suggested that miR-99b-3p is an important component of skeletal muscle development.

Skeletal muscle development relies on the proliferation, differentiation, and regeneration of SMSCs to form new myofibers [15,18]. The myogenic regulatory factors, *MyoD* and *MyoG*, are the core components of the myogenic pathway, which play important roles during myogenesis and skeletal muscle differentiation [19]. *Pax7* is only expressed in satellite cells and is involved in regulating the self-renewal of adult skeletal muscle [20]. We found that the expression levels of *MyoG* and *Pax7* significantly increased after miR-99b-3p downexpression, while no significant difference was found with miR-99b-3p overexpression. Furthermore, we detected the expression level of miR-99b-3p during the differentiation process, and found that it first increased but then decreased, while the mRNA expression levels of *Caspase-3* and *NCOR1* were opposite to that of chi-miR-99b-3p. Additionally, the dual-luciferase reporter system confirmed the binding relationship between miR-99b-3p and Caspase-3, as well as *NCOR1*. These results indicated that miR-99b-3p might participate in the cell cycle regulation process of SMSCs. Additionally, we found that the expression levels of *MyoD* and *MyoG* gradually increased during the differentiation process (Figure S3), especially in the 7 d of SMSCs differentiation, when the expression of chi-miR-99b-3p decreased. However, whether chi-miR-99b-3p is related to the regulation of cell differentiation needs to be further explored.

Caspases are the main initiators of apoptosis and play crucial roles in mammals. *caspase-8*, *-9* and *-10* are initiator caspases, involved in the onset and amplification of apoptosis; *caspase-3*, *-6*, and *-7* are effector caspases, involved in the execution of apoptotic programs [21]. High expression of *caspase-3* was associated with augmented muscle protein catabolism and promoted apoptosis of SMSCs in hemodialysis patients [22]. Downregulation of *Caspase-3* protects denervation-induced muscle atrophy through inhibiting apoptosis [23]. Furthermore, *NCOR1* is a master regulator of mitochondrial metabolism in muscle, which is inversely associated with key mitochondrial and MyoGenic genes [24]. These studies found that *NCOR1*-null mice had increased muscle mass, which was accompanied by increases in the number and activity of mitochondria in muscle cells, and significantly enhanced exercise capacity [25]. In addition, a previous study found that miR-99b-3p promoted the proliferation of hepatocellular carcinoma [8]. Here, we consistently found that miR-99b-3p inhibited the expressions of *caspase-3*, *-7*, *-9*, and *BCL2*, which promoted the intrinsic apoptotic pathway [26]. In addition, CCK-8 assays showed that the miR-99b-3p mimic promoted cell proliferation, while the miR-99b-3p inhibitor suppressed cell proliferation. *Caspase-3* and *NCOR1* are direct targets of miR-99b-3p. Thus, our findings indicated that miR-99b-3p might act as a “gatekeeper” for the intrinsic apoptotic pathway in skeletal muscle, which provides further insights into the mechanisms underlying the regulation of intrinsic skeletal muscle apoptosis.

To further investigate the detailed regulatory role of miR-99b-3p in skeletal muscle, RNA sequencing was used to assess the integrated miRNA–mRNA network changes after miR-99b-3p upregulation in SMSCs. We identified that the DE genes were enriched in the pathways associated with cell cycle, relaxin signaling, DNA replication, and protein digestion and absorption. Among them, the cell cycle pathway was the most enriched pathway, and the 48 DE genes were screened to participate in this pathway. Most of the DE genes were significantly downregulated in SMSCs after miR-99b-3p upregulation, including the pro-apoptosis-related gene *BCL2*, further suggesting that overexpression of miR-99b-3p can inhibit apoptosis in SMSCs. However, we did not find any downregulation of *Caspase-3* or *NCOR1* in SMSCs after miR-99b-3p overexpression by sequencing, which might have been due to the limitation of sequencing depth.

Additionally, we found that several miRNAs, including miR-199c-3p, miR-199a-3p, let-7c-3p, let-7c-5p, miR-10a-5p, miR-125b-3p, and miR-99a-5p, were highly expressed in SMSCs after miR-99b-3p upregulation, and these have been reported to enhance muscle regeneration [27,28,29,30] or promote cell proliferation in some cancers [31,32,33]. Furthermore, miR-199c-3p, miR-199a-3p, miR-99a-3p, and miR-99b-3p belong to the same miRNA cluster, suggesting that overexpression of miR-99b-3p induces the expression of a series of cell cycle regulation related miRNAs, further promoting cell proliferation and differentiation in SMSCs. Subsequently, KEGG enrichment analysis of these DE miRNAs revealed that most of the DE genes were predicted to be targeted by the DE miRNAs and were involved in the cell cycle process, further confirming a role for miR-99b-3p in SMSCs.

Although we identified potential miRNA–mRNA interactions regulated by miR-99b-3p and involved in goat skeletal muscle development, it is worth noting that further experimental studies are required to validate these miRNA–mRNA interactions in goat skeletal development.

## 5. Conclusions

The results of this preliminary study confirmed that chi-miR-99b-3p is an miRNA associated with the proliferation and differentiation of goat SMSCs. Overexpression of miR-99b-3p promoted SMSC proliferation by targeting *Caspase-3* and *NCOR1* and inhibiting the expression of apoptosis-related genes. Upregulation of miR-99b-3p promoted the expression of several miRNAs including miR-199c-3p, miR-199a-3p, let-7c-3p, let-7c-5p, miR-10a-5p, miR-125b-3p, and miR-99a-5p, which are closely related to differentiation, proliferation, and apoptosis. These results suggested that miR-99b-3p may regulate the differentiation, proliferation, and apoptosis of SMSCs through *Caspase-3* and *NCOR1* in goats.

## Data Availability

The results from data analyses performed in this study are included in this article and the tables. The raw sequencing data were deposited into the NCBI SRA database with the free accession number PRJNA844520.

## Supplementary Materials

The following supporting information can be downloaded at: https://www.mdpi.com/article/10.3390/ani12182368/s1. Figure S1: *HDAC9* is not the target gene of chi-miR-99b-3p, (a) diagram depicting the binding sites of chi-miR-99b-3p on the 3′-UTR of *HDAC9*, (highlighted in red), (b) detection of relative luciferase activity; Figure S2: Original figures for figure 3a; Figure S3: mRNA expressions of selected targets during the differentiation of SMSCs; Table S1: Mimic/inhibitor sequence used in this study; Table S2: Details of primer sequence used in this study; Table S3: Potential targets of chi-miR-99b-3p involved in the regulation of skeletal muscle development; Table S4: DE genes associated with cell-cycle pathway; Table S5: Potential targets of miR-99b-3p screened by DE genes; Table S6: List of DE miRNA in SMSCs after chi-miR-99b-3p upregulation; Table S7: Overlapping genes screened by DE genes and predicted targeted of DE miRNAs.
