# Supplementary material for: chi-miR-99b-3p Regulates the Proliferation of Goat Skeletal Muscle Satellite Cells In Vitro by Targeting Caspase-3 and NCOR1
Source: Animals (Basel). 2022 Sep 11;12(18):2368. doi: 10.3390/ani12182368 (PMC9495177; doi:10.3390/ani12182368)
Supplement: Supplementary file 1 [file animals-12-02368-s001.zip › Supplementary FigureS1-S3.pdf]

Supplementary Figures

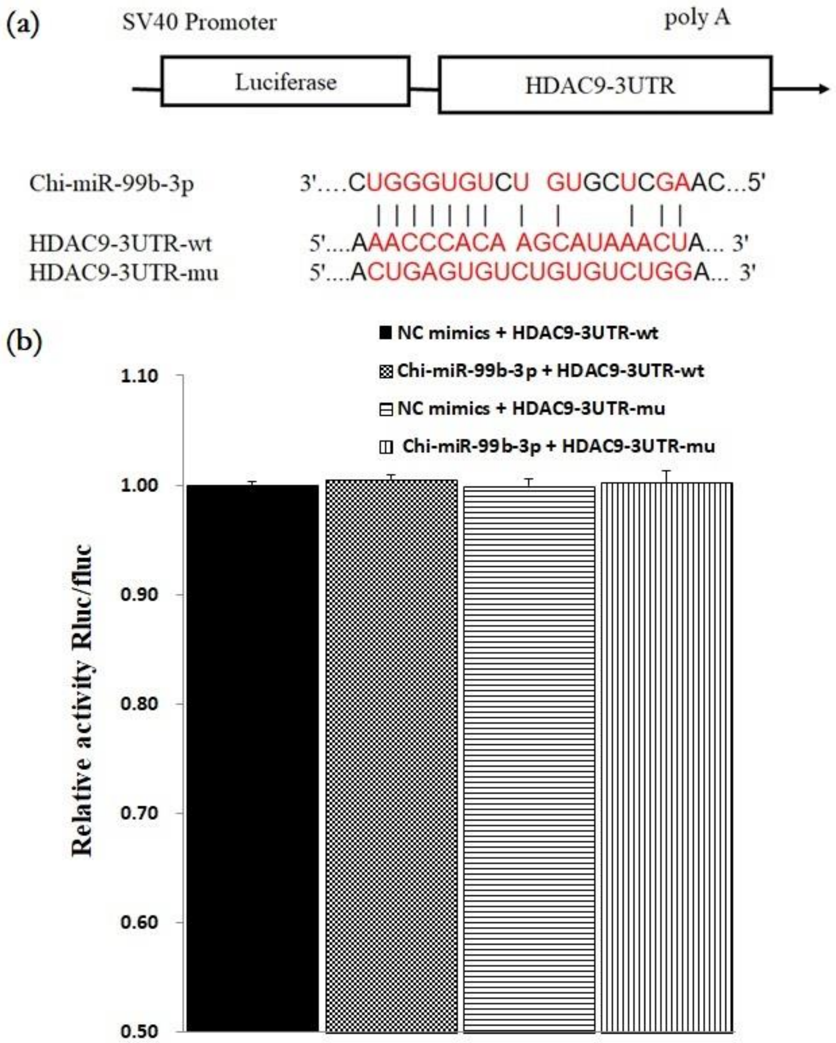

Figure S1. *HDAC9* is not the target genes of chi-miR-99b-3p, (a) Diagram depicting the binding sites of chi-miR-99b-3p on the 3′UTR of *HDAC9*, (highlighted in red). (b) Detection of relative luciferase activity. wt, wild-type. mut, mutant-type.

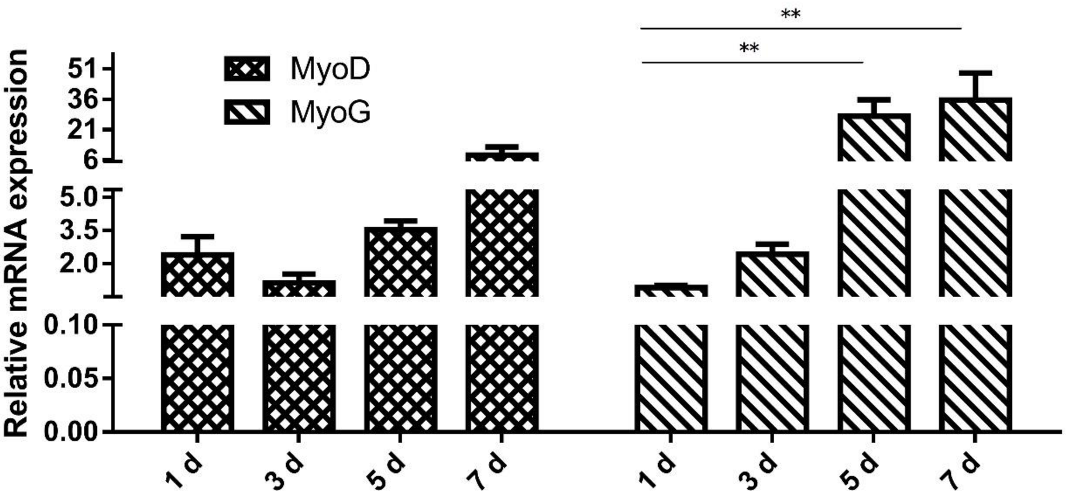

Figure S2. The mRNA expressions of selected targets during the differentiation of SMSCs.

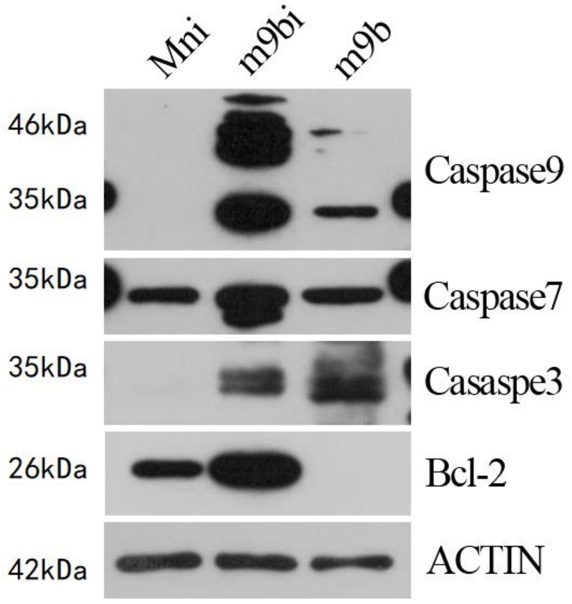

Figure 3a

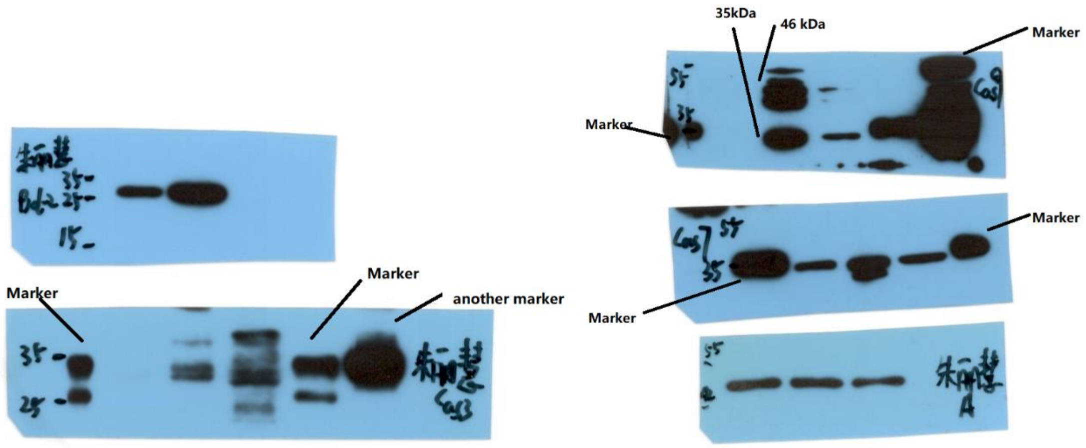

Figure S3. mRNA expressions of selected targets during the differentiation of SMSCs.
